# Supplementary material for: Observation of non-superconducting phase changes in nitrogen doped lutetium hydrides
Source: Nat Commun. 2023 Sep 26;14:5991. doi: 10.1038/s41467-023-41777-7 (PMC10522599; doi:10.1038/s41467-023-41777-7)
Supplement: Supplementary file 1 — Supplementary Information [file 41467_2023_41777_MOESM1_ESM.pdf]

Supplementary Information for

Observation of non-superconducting phase changes in nitrogen doped lutetium hydrides

Xiangzhuo Xing[1,2#], Chao Wang[1,2#], Linchao Yu[1], Jie Xu[1], Chutong Zhang[1], Mengge Zhang[1], Song Huang[1], Xiaoran Zhang[1], Yunxian Liu[1,2], Bingchao Yang[1,2], Xin Chen[1,2], Yongsheng Zhang[1,2], Jian-gang Guo[3], Zhixiang Shi[4], Yanming Ma[5,6,7], Changfeng Chen[8] & Xiaobing Liu[1,2*]

[1]Laboratory of High Pressure Physics and Material Science (HPPMS), School of Physics and Physical Engineering, Qufu Normal University, Qufu 273165, China

[2]Advanced Research Institute of Multidisciplinary Sciences, Qufu Normal University, Qufu 273165, China

[3]Beijing National Laboratory for Condensed Matter Physics, Institute of Physics, Chinese Academy of Sciences, Beijing 100190, China

[4]School of Physics, Southeast University, Nanjing 211189, China

[5]Innovation Center for Computational Methods & Software, College of Physics, Jilin University, Changchun 130012, China

[6]State Key Laboratory of Superhard Materials, Jilin University, Changchun 130012, China

[7]International Center of Future Science, Jilin University, Changchun 130012, China

[8]Department of Physics and Astronomy, University of Nevada, Las Vegas, Nevada 89154, USA

[#] These authors contributed equally: Xiangzhuo Xing, Chao Wang.

[*] Corresponding author. e-mail: xiaobing.phy@qfnu.edu.cn

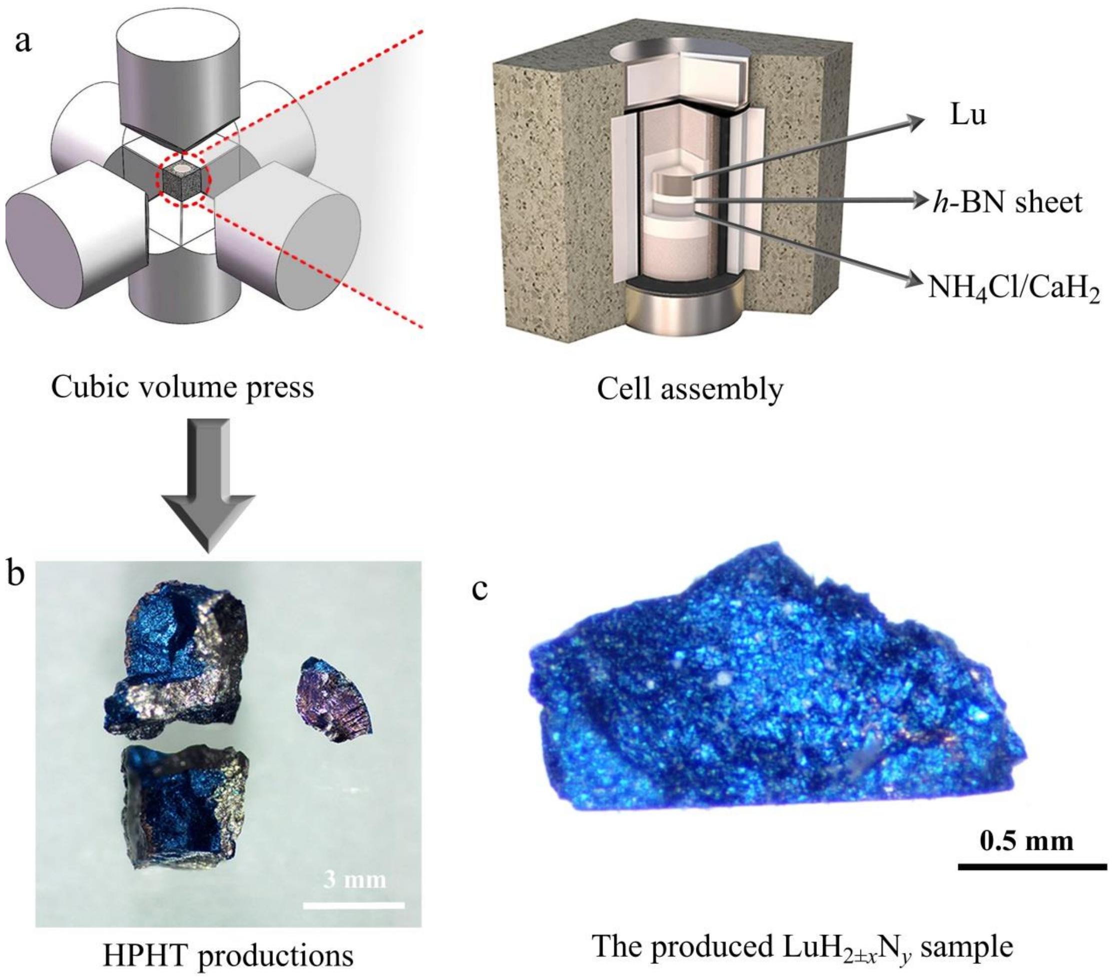

Supplementary Fig. 1 | Sample synthesis. a, Schematic illustration of the employed cubic-type high pressure apparatus and HPHT chamber for the synthesis of $LuH_{2\pm x}N_y$ samples. **b**, Optical images of the HPHT products. Most of the as-grown sample surfaces are in silver color. By cracking the sample, the interior shows a uniform shining blue color. **c**, The blue color of the synthesized sample interior is very similar to that of the previously reported $LuH_{3-\delta}N_\varepsilon$ at ambient pressure[1].

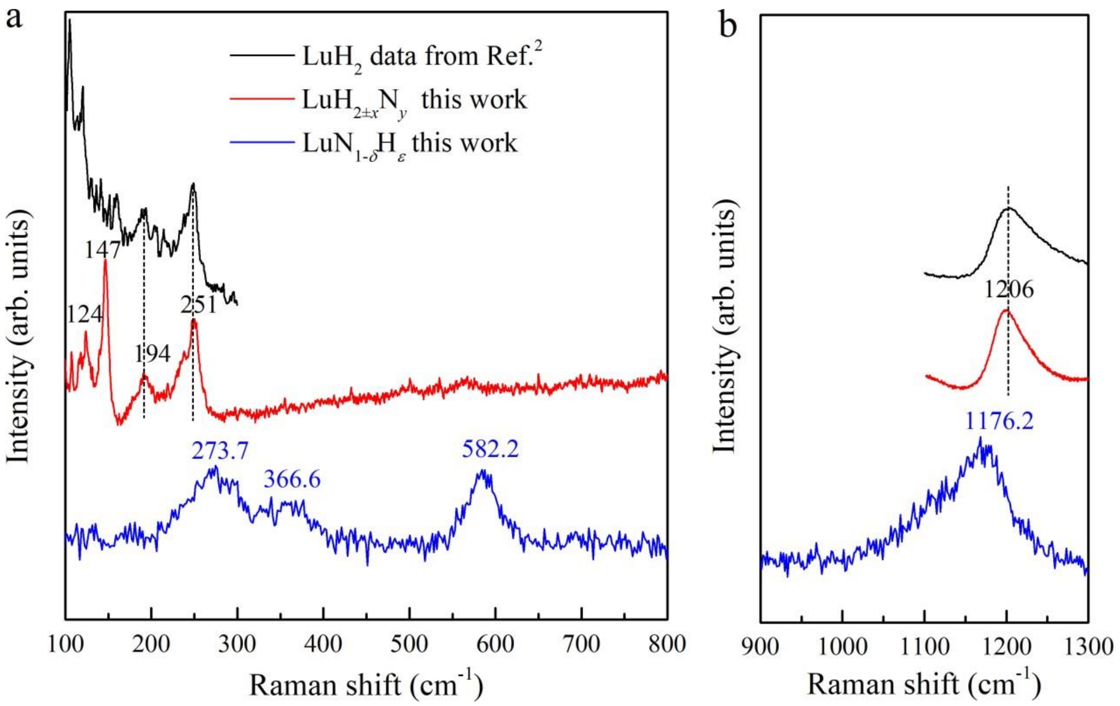

Supplementary Fig. 2 | Typical Raman spectra of $LuH_{2\pm x}N_y$ and $LuN_{1-\delta}H_\varepsilon$ samples. a and **b**, Comparison of Raman spectra for our produced $LuH_{2\pm x}N_y$ and $LuN_{1-\delta}H_\varepsilon$ samples at low and high wavenumber regions, respectively. Data on LuH_2 from Ref.[2] are plotted as black lines for comparison. The $LuN_{1-\delta}H_\varepsilon$ bulk samples were synthesized by HPHT method with the same experimental setup and pressure/temperature conditions as the synthesis of $LuH_{2\pm x}N_y$ samples in this work. The starting materials are LuN powders (claimed as 99.999 *wt*.% in purity, but detected certain hydrogen doping in our laboratory). The mixture of NH_4Cl and CaH_2 was used as the source of nitrogen and hydrogen for the HPHT synthesis. The experimental details for synthesis of $LuN_{1-\delta}H_\varepsilon$ sample are given in the description of $LuH_{2\pm x}N_y$ samples and Supplementary Fig. 1. Both the raw materials and produced $LuN_{1-\delta}H_\varepsilon$ samples show similar Raman spectra with characteristic peaks at 273.7 cm^{-1}, 366.6 cm^{-1}, 582.2 cm^{-1}, and 1176.2 cm^{-1}. It is clearly seen that the hydrogen-related vibrational modes at high wavenumbers of 1176.2 cm^{-1} show about 30 cm^{-1} deviation from that of LuH_2 and $LuH_{2\pm x}N_y$, while the characteristic peak of LuN[3] at 582.2 cm^{-1} and other peaks are completely absent in the $LuH_{2\pm x}N_y$ samples of this work and also in that of Ref.[1]. Moreover, we notice that the Raman peaks at lower wavenumbers of 124 cm^{-1} and 147 cm^{-1} only appear in the $LuH_{2\pm x}N_y$ samples, but are absent in the $LuN_{1-\delta}H_\varepsilon$ and the LuH_2 [2] samples. This result strongly indicates that no $LuN_{1-\delta}H_\varepsilon$ phase is present in our synthesized $LuH_{2\pm x}N_y$ samples.

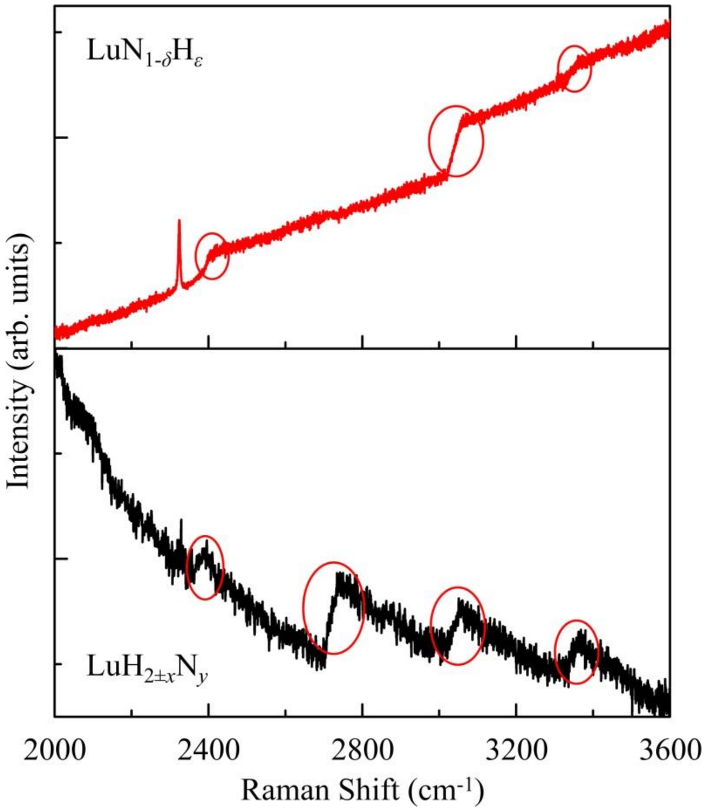

Supplementary Fig. 3 | Typical Raman spectra of $LuH_{2\pm x}N_y$ and $LuN_{1-\delta}H_\varepsilon$ samples at high wavenumber region. Considering the N-H stretching region is usually shown in the high wavenumber region, we measured the Raman spectra in the 2000-3600 cm^{-1} region. No N-H related stretching modes can be detected throughout the whole measured region, indicating the small amount of NH species. Only a sharp peak for the possible N-N related stretching mode[4] at 2328 cm^{-1} and 2324.5 cm^{-1} can be observed in two samples, possibly attributing to some N_2 chemisorbed on the sample surface in the air. These marked jumps in the red cycles are resulted from the grating change during the Raman measurements.

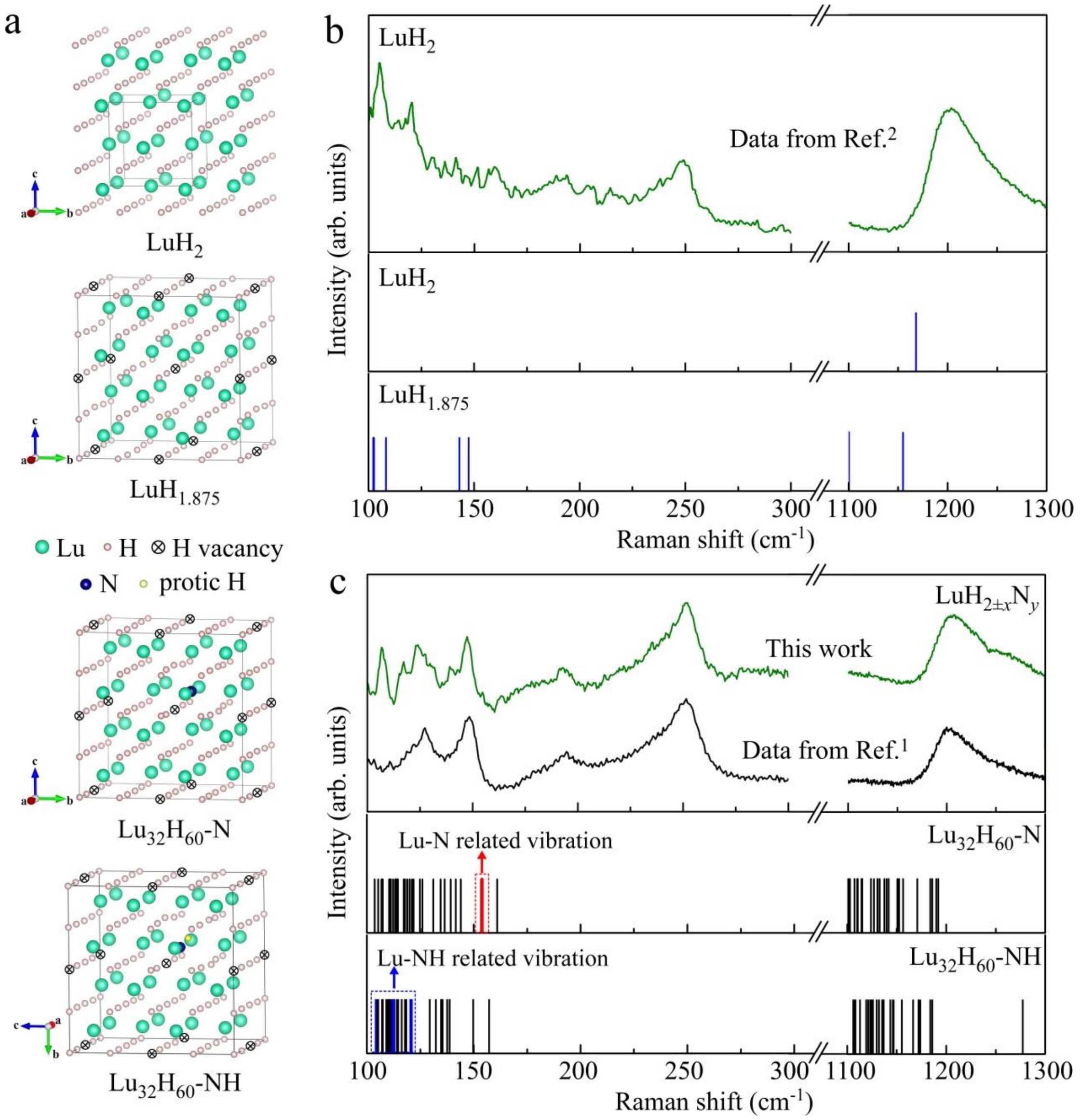

Supplementary Fig. 4 | Theoretical simulation of crystal structure and Raman spectra. a, Structures of the pristine LuH_2 compound, with H vacancies ($LuH_{1.875}$), and plus one N interstitial ($Lu_{32}H_{60}$-N) and one NH cluster ($Lu_{32}H_{60}$-NH) nearby vacancies (from top to bottom). **b** and **c**. Comparison of experimental and calculated Raman spectra. Since the cubic structure with $Fm\bar{3}m$ space group has high symmetry, low energy Raman modes arising from the reduced symmetry of the LuH_2 framework are induced by the H vacancies in the lattice. The Raman modes become rich and strong when N atoms are incorporated in $Lu_{32}H_{60}$-N, resulting in further reduction of the symmetry of the structure, consistent with the experimental results. Interestingly, a Lu-N related vibration at 154 cm^{-1} (marked as red line) in $Lu_{32}H_{60}$-N contributes to the presence of the modes at 147 cm^{-1} in the measured $LuH_{2\pm x}N_y$ samples[1]. The incorporated NH species in $Lu_{32}H_{60}$-NH play significant roles in the lower wavenumber region, leading to the appearance of three Raman

vibrations as 104.5 cm^{-1}, 112.3 cm^{-1}, and 120.6 cm^{-1} (marked as blue lines), consistent with the observation of a broad band at 124 cm^{-1} in the measured Raman spectra of $LuH_{2\pm x}N_y$ samples[1]. While the origin of the two peaks at 194 cm^{-1} and 251 cm^{-1} in the measured $LuH_{2\pm x}N_y$ samples (both shown in recently reported LuH_2 samples[2]) remains uncertain, it is reasonable to attribute them to other factors inducing the reduction of structural symmetry by vacancies related defects or interstitial hydrogen atoms.

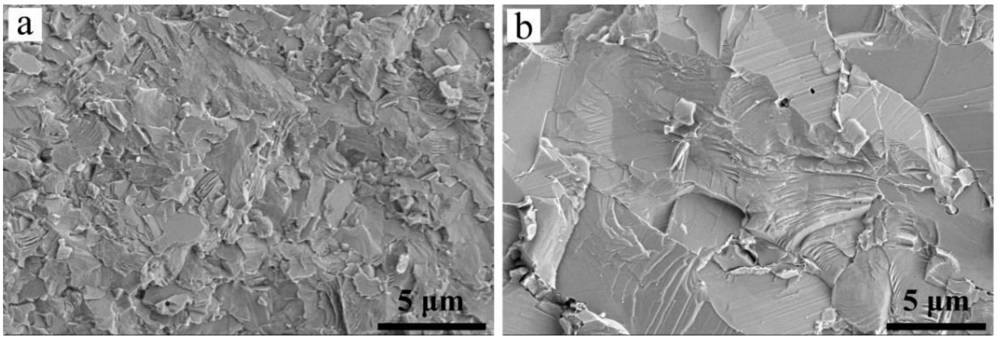

Supplementary Fig. 5 | Microstructure of the produced $LuH_{2\pm x}N_y$ samples. a and **b**, Typical scanning electron microscope images of the $LuH_{2\pm x}N_y$ samples under ambient conditions.

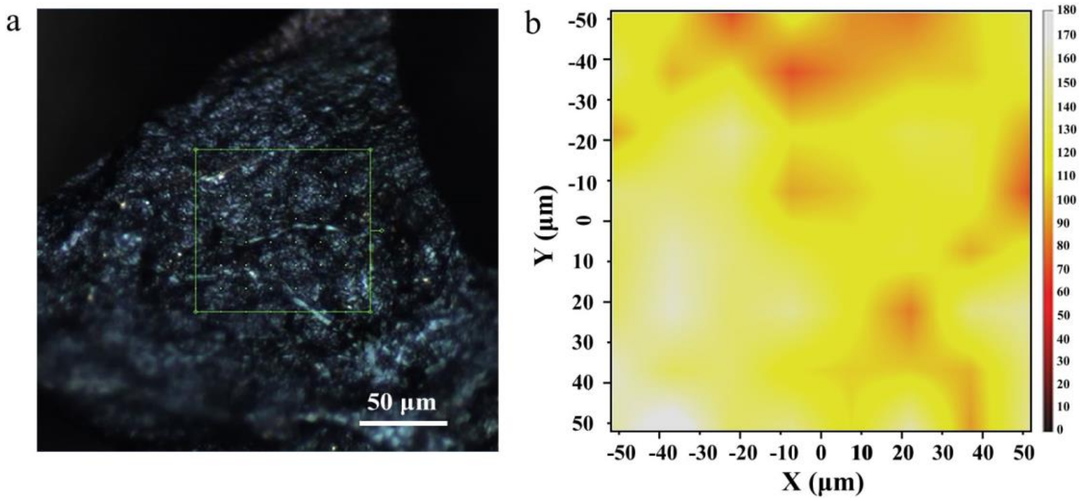

Supplementary Fig. 6 | Composition characterization for the produced $LuH_{2\pm x}N_y$ samples by Raman spectroscopy. a, Optical image of the area for mapping. **b**, Mapping data of the selected area shows a pure $LuH_{2\pm x}N_y$ phase.

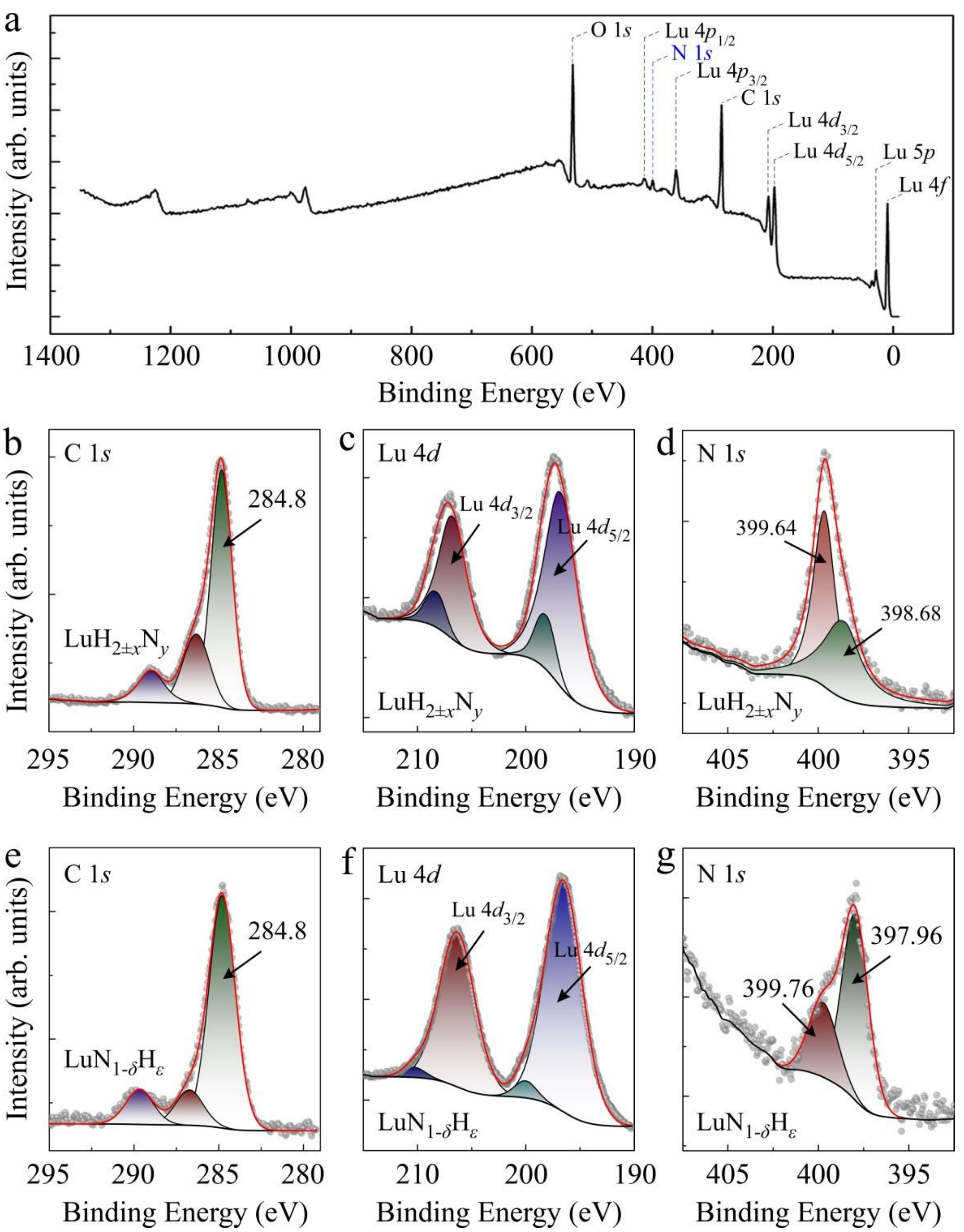

Supplementary Fig. 7 | Evidence of incorporation of nitrogen in the produced $LuH_{2\pm x}N_y$ and $LuN_{1-\delta}H_\varepsilon$ samples. a, Typical X-ray photoelectron spectroscopy (XPS) of the $LuH_{2\pm x}N_y$ samples, clearly indicating the presence of nitrogen in the produced samples. **b- d**, XPS spectra of C 1*s*, Lu 4*d* and N1*s* core levels of the $LuH_{2\pm x}N_y$ samples. The carbon phase (284.8 eV) from the XPS chamber is used for calibration. **e-g**, XPS spectra of C 1*s*, Lu 4*d* and N 1*s* core levels in the produced $LuN_{1-\delta}H_\varepsilon$ samples, respectively. The N 1*s* core level composing of two binding energies at 397.96 eV and 399.76 eV, are attributed to the dominant N-Lu and partial N-H[5-7] bonding, respectively.

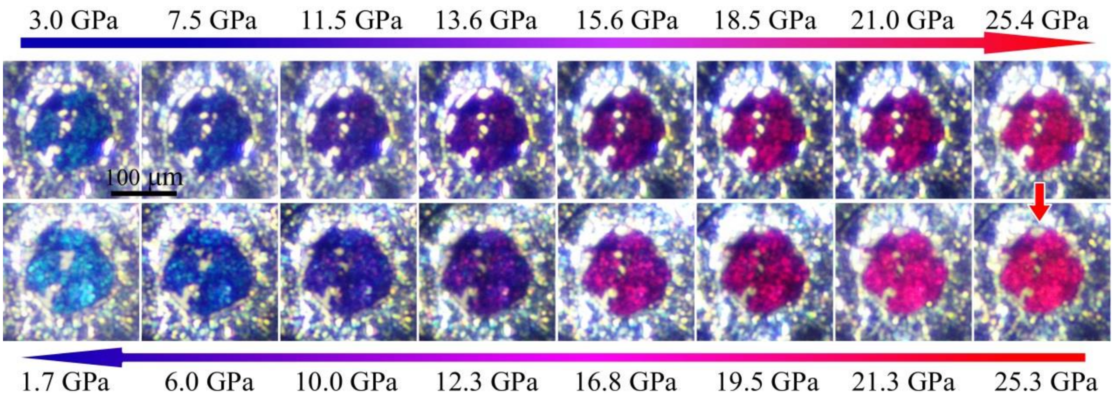

Supplementary Fig. 8 | Pressure-induced color change of the $LuH_{2\pm x}N_y$ sample-I compressed in nitrogen gas medium. Optical images of $LuH_{2\pm x}N_y$ sample during compression (top row) and decompression (bottom row). As rising pressure, the $LuH_{2\pm x}N_y$ sample undergoes a continuous color change from shinning blue at 0 GPa, to purplish blue at 11.5 GPa, to purple at 15.6 GPa, to pink at 18.5 GPa and to bright red at 25.4 GPa. After releasing pressure from 25.4 GPa to ambient pressure, the color gradually recovers to the original shinning blue.

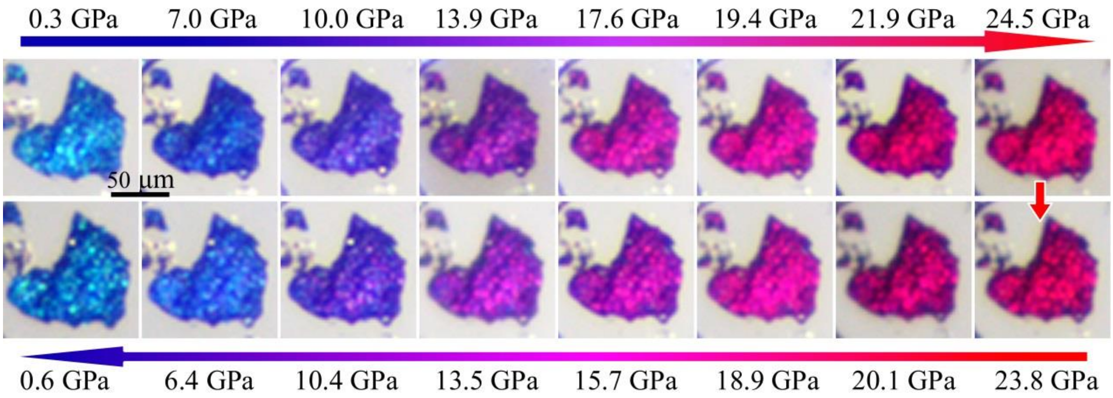

Supplementary Fig. 9 | Pressure-induced color change of the $LuH_{2\pm x}N_y$ sample-I compressed in water medium. Optical images of $LuH_{2\pm x}N_y$ sample during compression (top row) and decompression (bottom row). At rising pressure, the $LuH_{2\pm x}N_y$ sample undergoes a continuous color change from shinning blue at 0 GPa, to purplish blue at 10 GPa, to purple at 13.9 GPa, to pink at 17.6 GPa and to bright red at 21.9 GPa. After releasing pressure from 24.5 GPa to ambient pressure, the color gradually recovers to the original shinning blue.

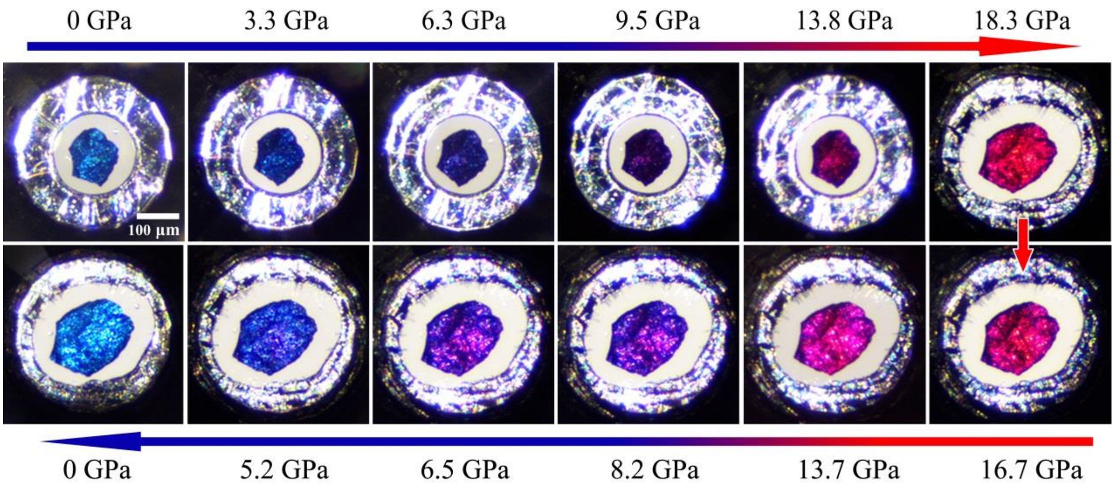

Supplementary Fig. 10 | Pressure-induced color change of the $LuH_{2\pm x}N_y$ sample-I compressed in silicone oil medium. Optical images of $LuH_{2\pm x}N_y$ sample during compression (top row) and decompression (bottom row). At rising pressure, the $LuH_{2\pm x}N_y$ sample undergoes a continuous color change from shinning blue at 0 GPa, to purplish blue at 6.3 GPa, to purple at 9.5 GPa, to pink at 13.8 GPa and to bright red at 18.3 GPa. After releasing pressure from 18.3 GPa to ambient pressure, the color gradually recovers to the original shinning blue.

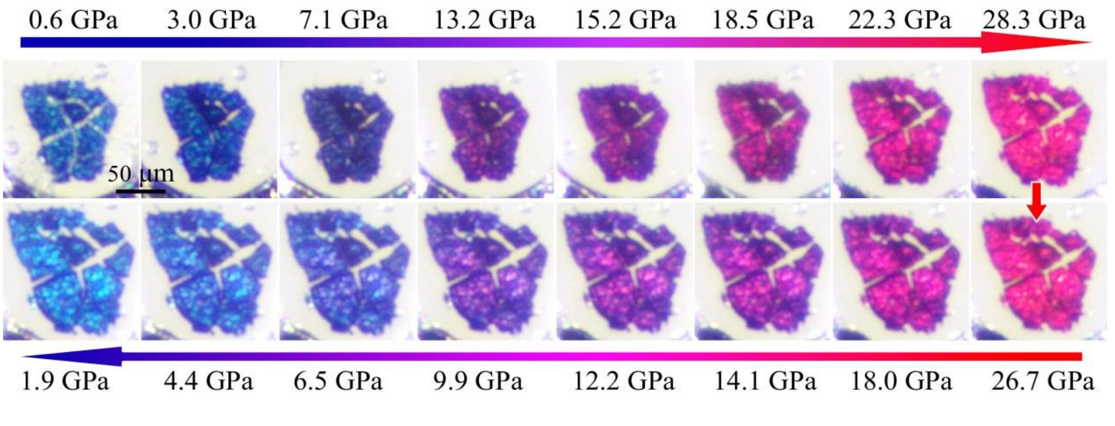

Supplementary Fig. 11 | Pressure-induced color change of the $LuH_{2\pm x}N_y$ sample-I compressed in NaCl medium. Optical images of $LuH_{2\pm x}N_y$ sample during compression (top row) and decompression (bottom row). At rising pressure, the $LuH_{2\pm x}N_y$ sample undergoes a continuous color change from shinning blue at 0 GPa, to purplish blue at 7.1 GPa, to purple at 13.2 GPa, to pink at 18.5 GPa and to bright red (28.3 GPa). After releasing pressure from 28.3 GPa to ambient pressure, the color gradually recovers to the original shinning blue.

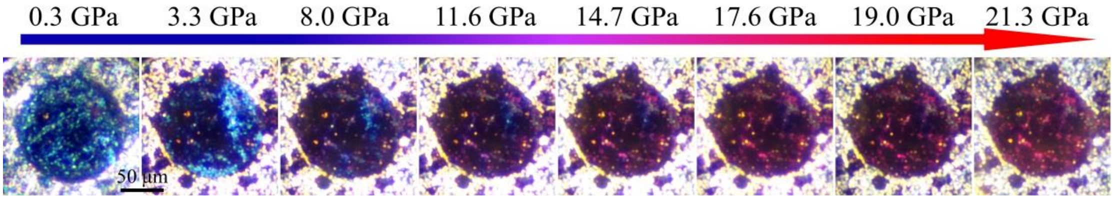

Supplementary Fig. 12 | Pressure-induced color change of the $LuH_{2\pm x}N_y$ sample-I compressed without any pressure medium. Optical images of $LuH_{2\pm x}N_y$ sample during compression (top row) and decompression (bottom row). A mixed color change with rising pressure is visible because of the inhomogeneous pressure distribution caused by the large pressure gradients in the DAC sample chamber.

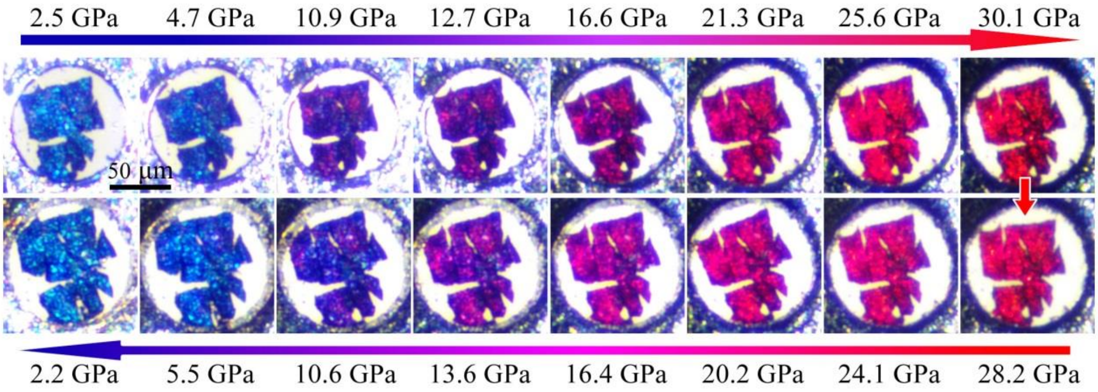

Supplementary Fig. 13 | Pressure-induced color change of $LuH_{2\pm x}N_y$ sample-II compressed in nitrogen gas medium. Optical images during compression (top row) and decompression (bottom row). At rising pressures, the $LuH_{2\pm x}N_y$ sample undergoes a continuous color change from shinning blue at 0 GPa, to purplish blue at 10.9 GPa, to purple at 16.6 GPa, to pink at 21.3 GPa and to bright red at 25.6 GPa. After releasing pressure from 30.1 GPa to ambient pressure, the color gradually recovers to the original shinning blue.

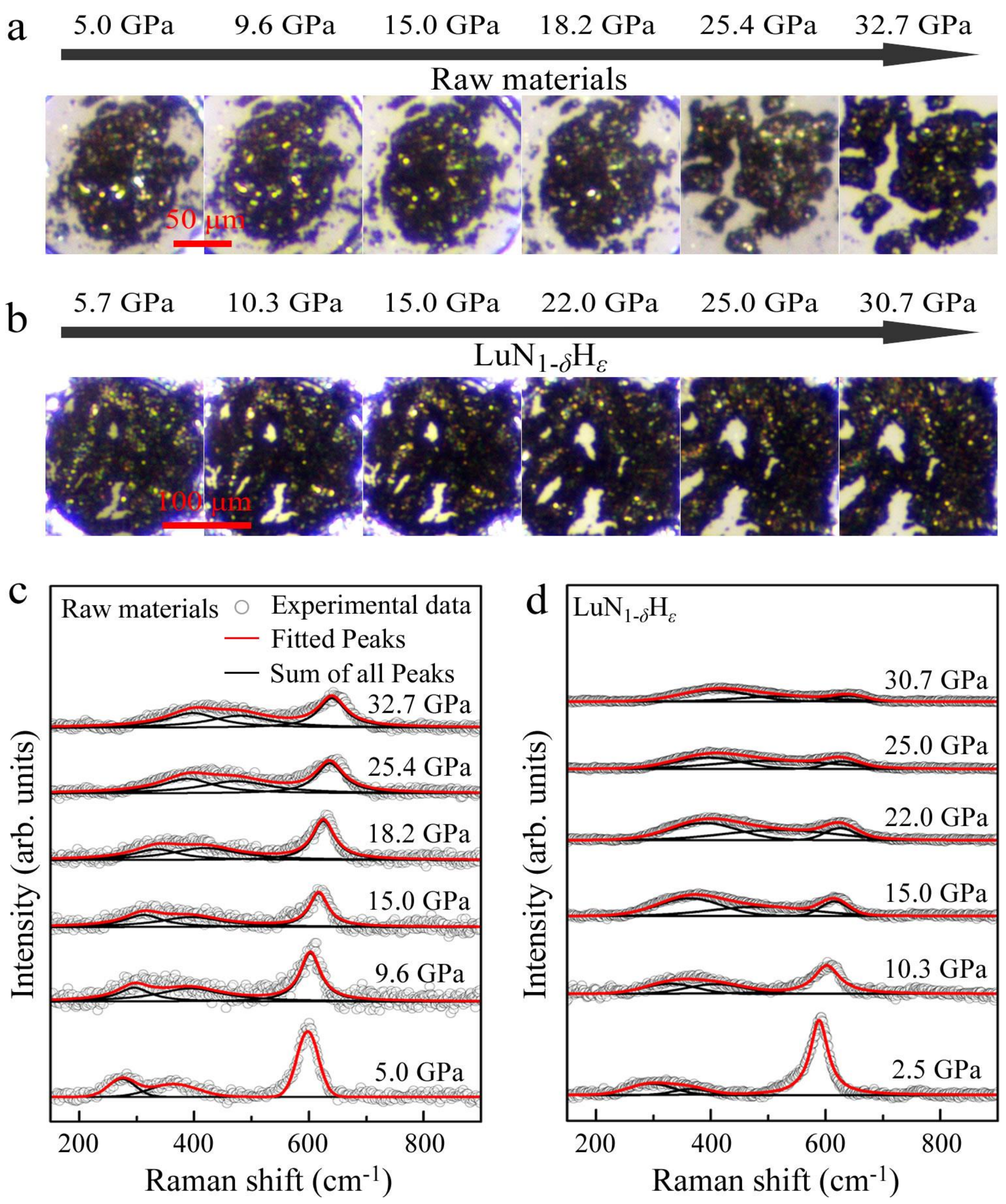

Supplementary Fig. 14 | Optical images and Raman spectra of raw LuN materials and the produced $LuN_{1-\delta}H_{\varepsilon}$ samples under pressure. a and **b**, Typical optical images of raw LuN materials and the produced $LuN_{1-\delta}H_{\varepsilon}$ samples at different pressures. As the pressure increases, the dark gray color of the sample remains unchanged. **c** and **d**, Pressure-dependent Raman spectra of raw LuN materials and the produced $LuN_{1-\delta}H_{\varepsilon}$ samples. The red lines represent the multi-peak fitting analysis.

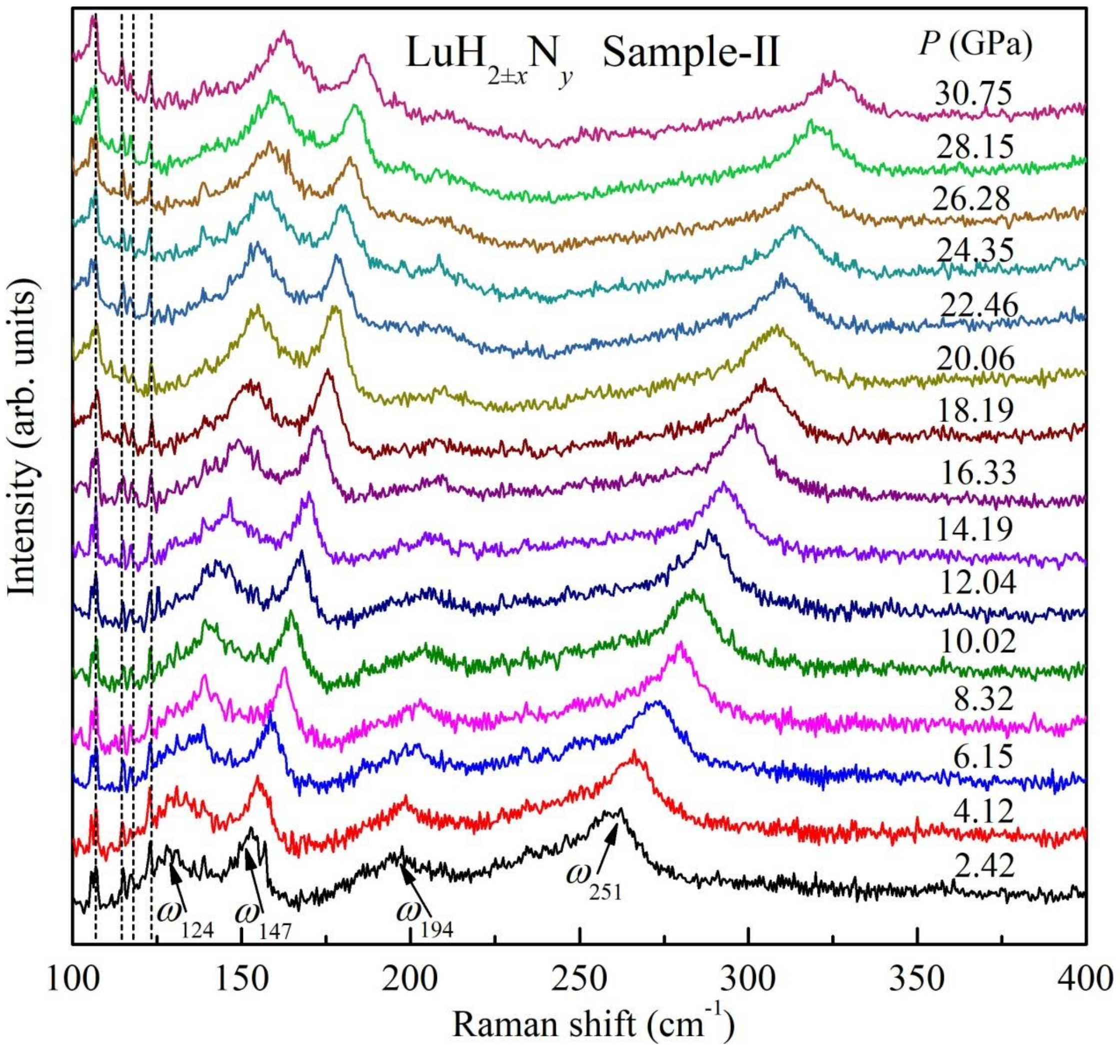

Supplementary Fig. 15 | *In-situ* Raman spectrum of $LuH_{2\pm x}N_y$ (sample-II) at changing pressures up to 30.75 GPa. The signals marked by dashed lines are from the noise of the Raman instrument, which do not shift with increasing pressure.

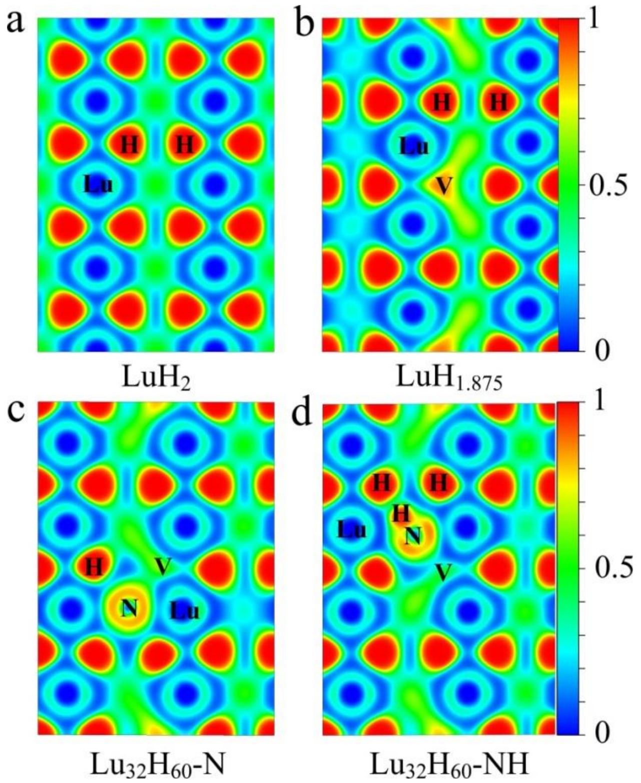

Supplementary Fig. 16 | Electron distribution in lutetium hydride. The calculated electron localization function (ELF) at 0 GPa for pristine LuH_2 (**a**), with H vacancies $LuH_{1.875}$ (**b**), and plus one N interstitial $Lu_{32}H_{60}$-N (**c**) and one NH cluster $Lu_{32}H_{60}$-NH (**d**). It is clear from (a) and (b) that in $LuH_{1.875}$ at the ambient condition, electrons from Lu atom are transferred to neighboring H atoms and H vacancy. The ELF value at the H vacancy (V_H) position is ~0.88, indicating the presence of highly localized electrons. With the introduction of N atom near the H vacancy ($Lu_{32}H_{60}$-N), the N atom sucks the localized electrons around the H vacancy and delocalizes them at V_H (ELF=~0.64). With the introduction of NH cluster near the H vacancy ($Lu_{32}H_{60}$-NH), the N-H form covalent bonds in the NH cluster, and the ELF value at the H vacancy (V_H) position is about 0.48. Additionally, when pressure is applied, the distance of N-V_H decreases, and the effects of N and NH on electron redistribution is more noticeable. The NH/N induced electron redistribution is consistent with our experimental results presented in Fig. 3c, which show a significant effect on NH/N-related Raman peaks at 124 cm^{-1} and 143 cm^{-1} with increasing pressure in the pink phase region. Overall, the calculations reveal that the incorporation of N atom plays an important role in altering chemical bonding and electron distribution in the LuH_2 framework, thus contributing to the change of intrinsic physical properties, such as the electrical conductivity and the color of sample.

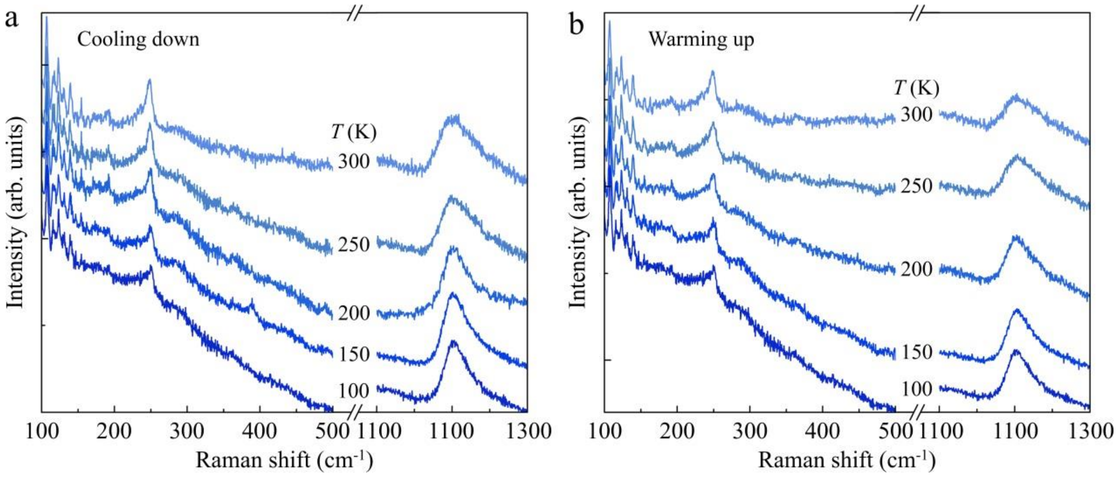

Supplementary Fig. 17 | Raman spectra of $LuH_{2\pm x}N_y$ at different temperatures below 300 K. **a** and **b**, Raman spectrum collected during the cooling down and warming up processes at ambient pressure, in which no obvious change is observed.

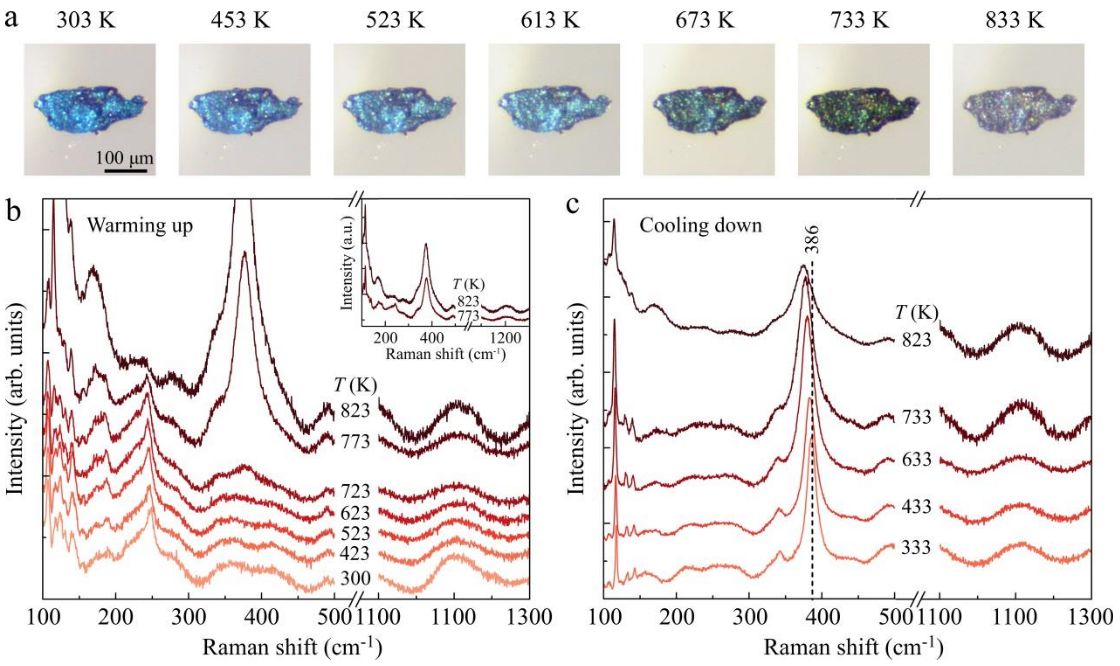

Supplementary Fig. 18 | Color change and Raman spectra at high temperatures above 300 K. **a**. Optical images of $LuH_{2\pm x}N_y$ sample with increasing temperature at ambient pressure. As clearly seen, a temperature-induced color change occurs, from shining blue at room temperature to green at 733 K. **b** and **c**, Temperature-dependent Raman spectra collected during the warming up and cooling down processes. As temperature increases, a new peak with high intensity appears at around 386 cm^{-1} accompanying with the green phase, which remains when temperature is lowered to room temperature.

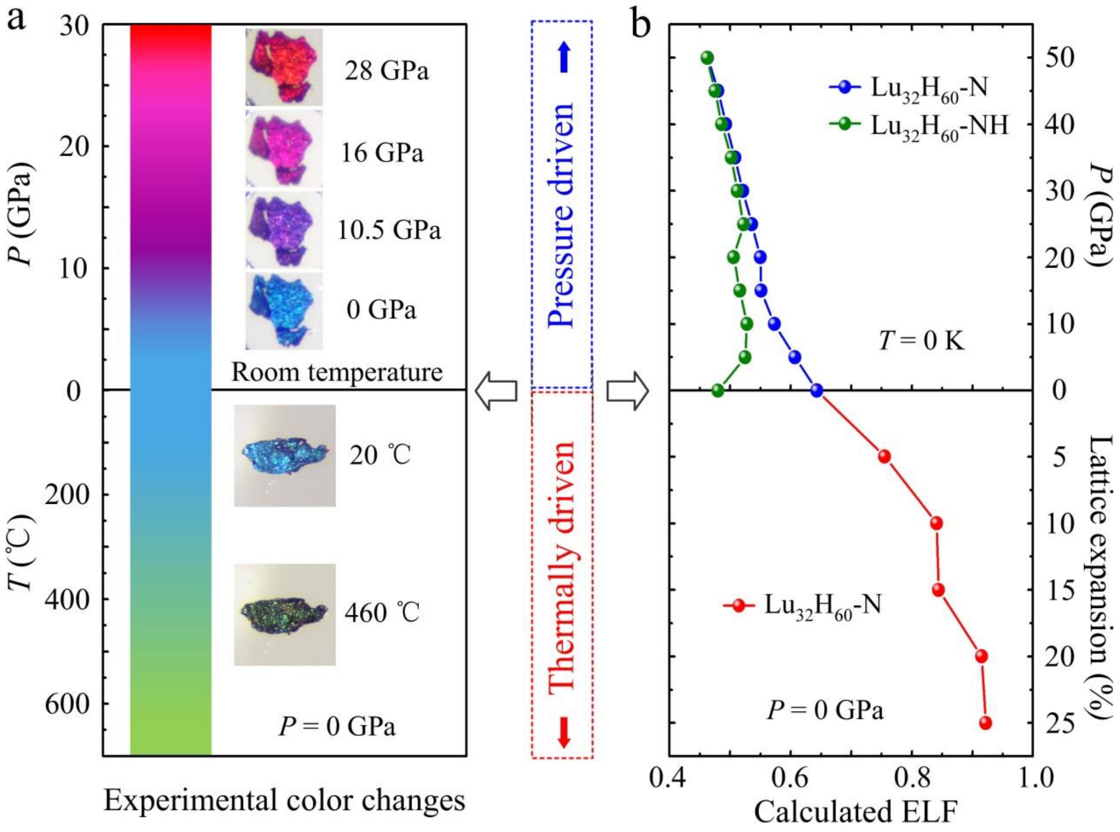

Supplementary Fig. 19 | Pressure and thermally driven color change and ELF in lutetium hydride. a, The experimental observation of color changes in our produced $LuH_{2\pm x}N_y$ samples as a function of high pressure (top) and temperature (bottom). **b**, Correspondence of the calculated ELF value for the $Lu_{32}H_{60}$-N and $Lu_{32}H_{60}$-NH structures (Supplementary Fig. 4) under compression (top) and with lattice expansion state for the $Lu_{32}H_{60}$-N (bottom), simulating thermally driven effect on the constructed structure. The $Lu_{32}H_{60}$-NH structures induce thermal instability.

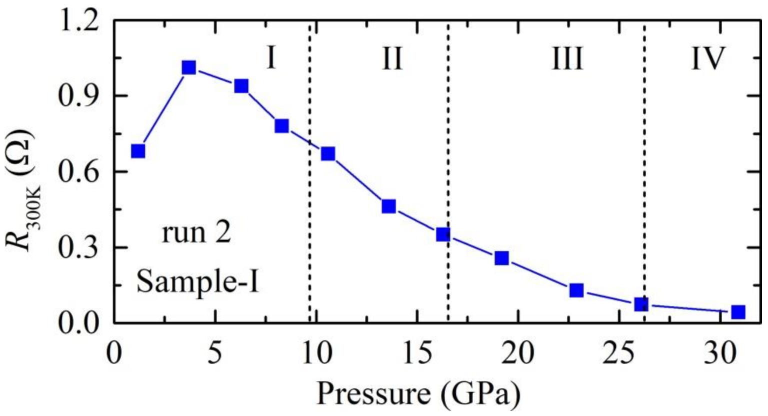

Supplementary Fig. 20 | R_{300K} versus Pressure. Evolution of the room-temperature resistance of $LuH_{2\pm x}N_y$ sample-I with pressure in run 2. Under rising pressure, the resistance first increases until pressure reaches 3.7 GPa, then gradually decreases up to pressure of 30.9 GPa.

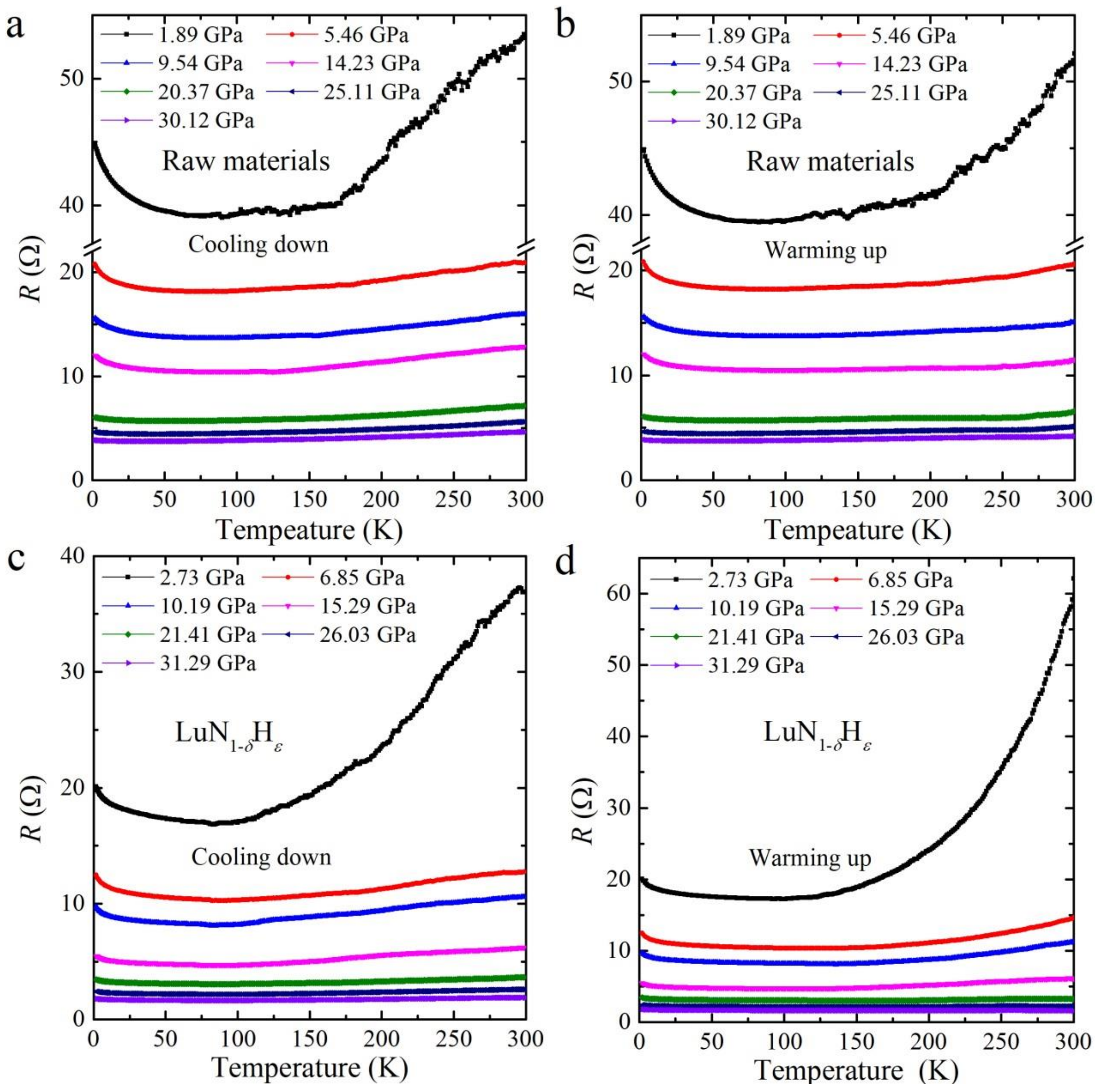

Supplementary Fig. 21 | Electrical transport measurements of raw LuN materials and the produced $LuN_{1-\delta}H_{\varepsilon}$ sample under pressure. a and **b**, Evolution of the resistance for the raw LuN materials with pressure during cooling down and warming up processes, respectively. **c** and **d**, Pressure-dependent evolution of the resistance for the $LuN_{1-\delta}H_{\varepsilon}$ sample, produced at 3 GPa and 773 K in a large volume press, during cooling down and warming up processes, respectively. The measured data show no sign of superconductivity in the raw LuN materials and our produced $LuN_{1-\delta}H_{\varepsilon}$ samples from 300 K to 1.8 K with increasing pressures, which has gone through all four phases of $LuH_{2\pm x}N_y$ samples (blue to red).

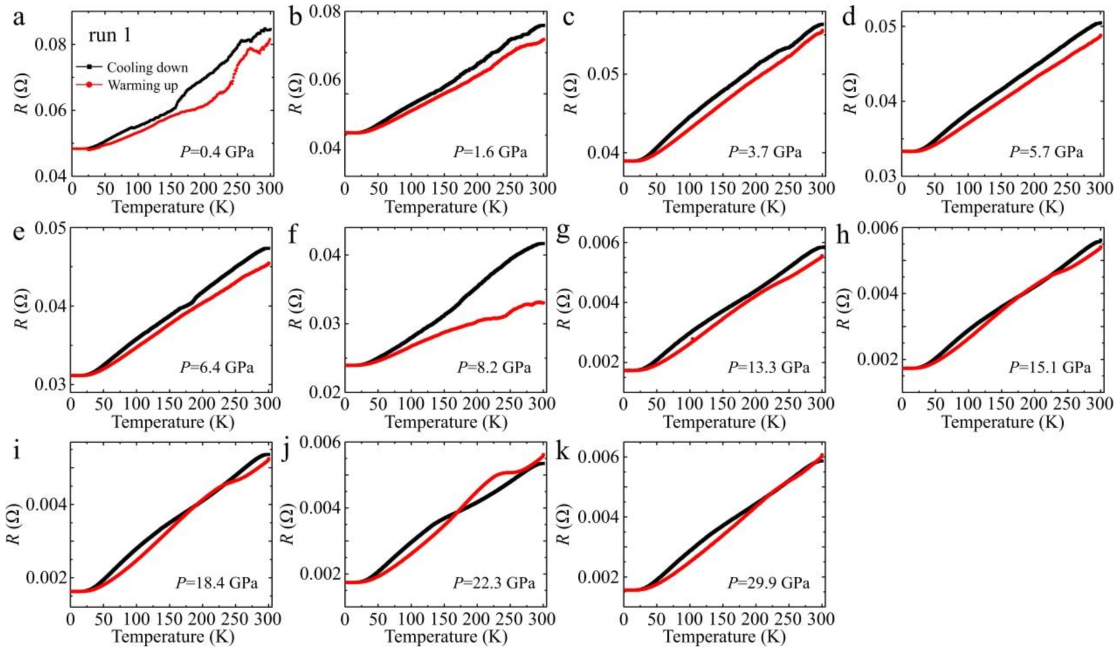

Supplementary Fig. 22 | Electrical transport measurements of sample-I under pressure for run 1. The temperature dependence of resistance of $LuH_{2\pm x}N_y$ for run 1 measured in the cooling-down and warming-up processes at different pressures: 0.4 GPa (**a**), 1.6 GPa (**b**), 3.7 GPa (**c**), 5.7 GPa (**d**), 6.4 GPa (**e**), 8.2 GPa (**f**), 13.3 GPa (**g**), 15.1 GPa (**h**), 18.4 GPa (**i**), 22.3 GPa (**j**) and 29.9 GPa (**k**). At P=8.2 GPa, the resistance in the cooling-down and warming-up processes starts to show different behaviors, followed by a large (an order of magnitude) drop at around 13.3 GPa, as described in the main text. At higher pressures above 13.3 GPa, a resistance kink at high temperatures is observed in the warming-up process. At low temperatures, a weak upturn is observed below 5.7 GPa, but is absent at higher pressures.

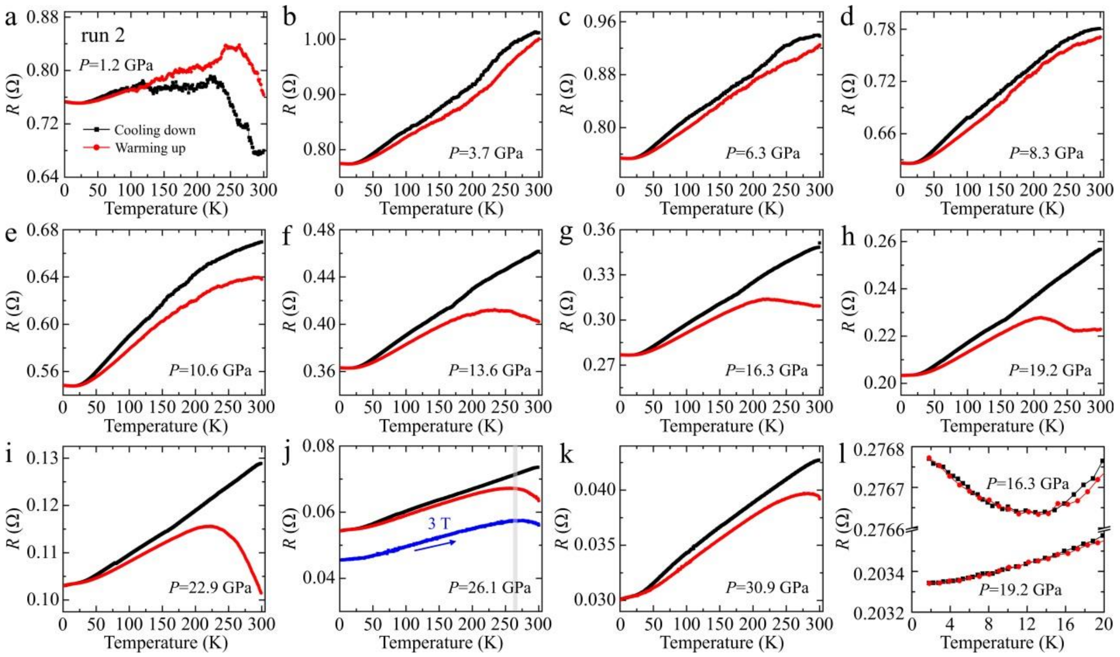

Supplementary Fig. 23 | Electrical transport measurements of sample-I under pressure for run 2. The temperature dependence of resistance of $LuH_{2\pm x}N_y$ for run 2 measured in the cooling-down and warming-up processes at different pressures: 1.2 GPa (**a**), 3.7 GPa (**b**), 6.3 GPa (**c**), 8.3 GPa (**d**), 10.6 GPa (**e**), 13.6 GPa (**f**), 16.3 GPa (**g**), 19.2 GPa (**h**), 22.9 GPa (**i**), 26.1 GPa (**j**) and 30.9 GPa (**k**). At *P*=1.2 GPa, the resistance displays a hump feature at high temperatures in both the cooling-down and warming-up processes. As the pressure increases up to 10.6 GPa, the resistance exhibits a monotonous behavior with changing temperature. With further increasing pressure, however, the resistance in the cooling-down and warming-up process exhibit contrasting behaviors, with an anomalous resistance hump emerging in the warming up process up to 30.9 GPa. (**l**) A weak resistance upturn is detected in the low temperature region at pressures below 16.3 GPa, which can be suppressed at higher pressures at or above 19.2 GPa. Moreover, to check whether the hump is related to a possible superconducting transition, a magnetic field was applied at *P* =26.1 GPa, and the results (**j**) show that the application of magnetic field did not move the peak of the resistance curve toward lower temperature as one would expect if the resistance drop was caused by the emergence of superconductivity in the sample. The shading vertical line is a guide to the eyes.

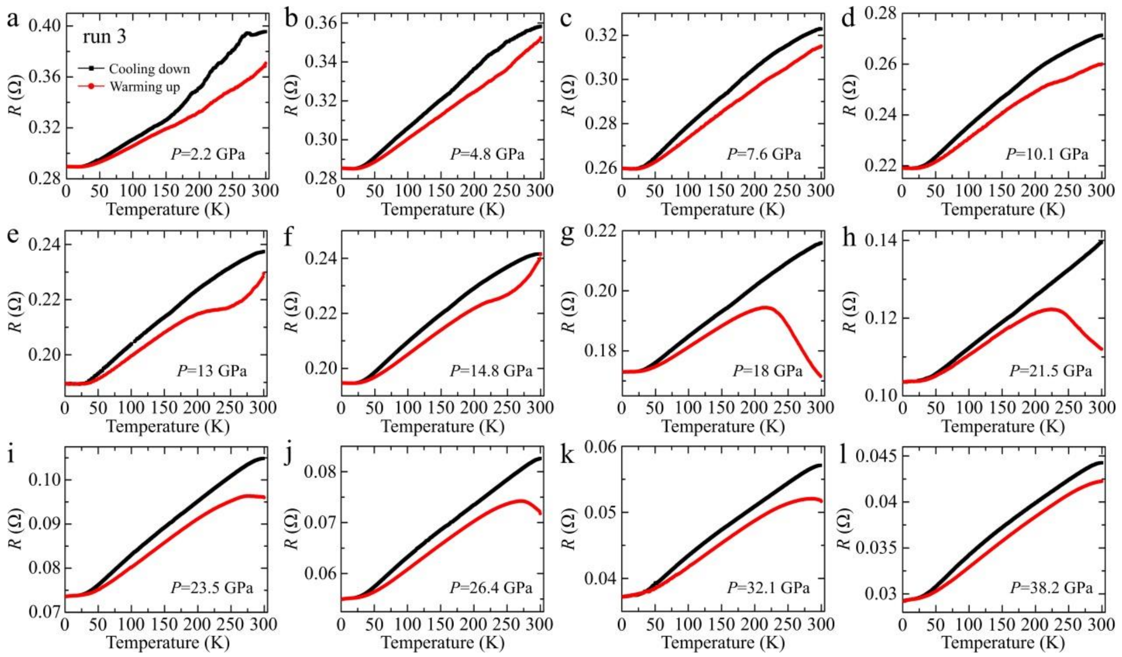

Supplementary Fig. 24 | Electrical transport measurements under pressure of sample-II in run 3. The temperature dependence of resistance of $LuH_{2\pm x}N_y$ in run 3 measured in the cooling-down and warming-up processes at different pressures: 2.2 GPa (**a**), 4.8 GPa (**b**), 7.6 GPa (**c**), 10.1 GPa (**d**), 13 GPa (**e**), 14.8 GPa (**f**), 18 GPa (**g**), 21.5 GPa (**h**), 23.5 GPa (**i**), 26.4 GPa (**j**), 32.1 GPa (k) and 38.2 GPa (**l**). At low pressures, the resistance shows similar behaviors with those seen in run 1 and run 2. It is noted that both the hump and plateau features that have seen in run 1 and run 2 in the resistance curve are also visible in this run, further indicating the same origin of the resistance anomaly.

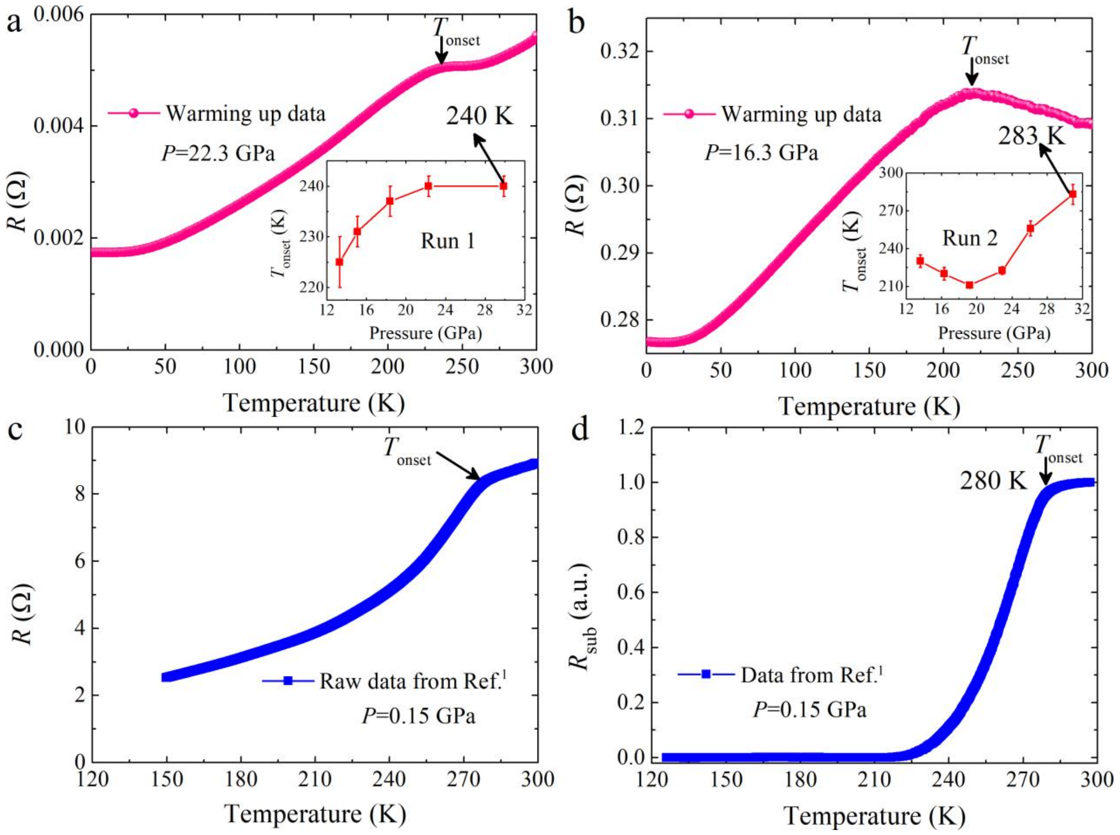

Supplementary Fig. 25 | Comparison of the resistance anomaly in $LuH_{2\pm x}N_y$ and the claimed superconducting transition[1]. As described in the main text, a resistance anomaly with a hump/plateau at high temperatures has been observed during the warming up process, with typical results shown in **a** and **b**. Such anomaly starts to appear in the purple phase and persists through the pink and red phases, possibly due to an electronic transition. As the temperature increases, the characteristic temperature T_{onset} labelled by the black arrows exhibits a rising trend in general through the pink phase, as shown in the insets of **a** and **b**. The error bars represent the uncertainty in determining T_{onset} by the minimum or the sign reversal of the derivative curve linear $dR(\mathrm{T})/dT$. It is seen that the warming-driven behavior of resistance anomaly below T_{onset} is very similar to that reported for near-ambient superconductors[1], especially for the pink phase at 22.3 GPa in our run 1. For comparison, the source data were downloaded from Ref. [1] and plotted in **c**, from which the superconducting state with zero resistance was achieved after subtracting a so-called background signal, as shown in **d**.

Supplementary Table 1. EDX data for the weight concentration of N and Lu elements in sample-I. Data 1-10 and 11-14 taken from randomly spots and mapping area of 50×35 μm in size, respectively.

Weight conc. (%) / Spot/Mapping number	Sample-I	
	N	Lu
1	0.09	99.91
2	0.11	99.89
3	0.78	99.22
4	0.30	99.70
5	0.28	99.72
6	0.05	99.95
7	0.03	99.97
8	0.15	99.85
9	0.05	99.95
10	0.08	99.92
11	0.04	99.96
12	0.23	99.77
13	0.28	99.72
14	0.30	99.70
Average	0.20	99.80

Supplementary Table 2. EDX data for the weight concentration of N and Lu elements in sample-II. All data taken from randomly spots.

Weight conc. (%) / Spot number	Sample II	
	N	Lu
1	0.36	99.64
2	1.36	98.64
3	0.15	99.84
4	0.17	99.83
5	0.73	99.27
6	0.26	99.74
7	0.26	99.74
8	0.27	99.73
9	2.78	97.22
10	0.14	99.86
11	2.44	97.56
12	0.84	99.16
13	1.02	98.98
14	1.58	98.42
15	0.31	99.69
Average	0.84	99.16

Supplementary References

1. Dasenbrock-Gammon, N. et al. Evidence of near-ambient superconductivity in a N-doped lutetium hydride. *Nature* **615**, 244 (2023).
2. Shan, P. et al. Pressure-induced color change in the lutetium dihydride LuH_2. *Chin. Phys. Lett.* **40**, 046101 (2023).
3. Granville, S. et al. Vibrational properties of rare-earth nitrides: Raman spectra and theory. *Phys. Rev. B* **79**, 054301 (2009).

4 Liu, C. et al. Raman Spectroscopy of Nitrogen Clathrate Hydrates. *Chin. J. Chem. Phys.***22**, 353 (2009).

5. Mozhchil, R. N. et al. Electronic, local atomic structure of lutetium tetraphenylporfyrin: XPS and XAFS spectroscopy stadies. *J. Phys.: Conf. Ser.* **1238**, 012002 (2019).
6. Asanova, T. I. et al. Studying the process of $(NH_4)_2[IrCl_6]$ thermal decomposition by X-Ray photoelectron spectroscopy and electron microscopy. *J. Struct. Chem.* **61**, 388-399 (2020).
7. Sitthiwet, C. et al. Hydrogen sorption kinetics and suppression of NH_3 emission of LiH-sandwiched $LiNH_2$-LiH-TiF_4-MWCNTs pellets upon cycling. *J. Alloy. Compd.* **909**, 164673 (2022).
